# Frequent attenders in primary health care in Finland: use of primary care services and patient characteristics

**DOI:** 10.1080/02813432.2026.2633757

**Published:** 2026-03-27

**Authors:** Kim Nygård, Jari Hartzell, Timo Kauppila, Essi Teronen, Ossi Rahkonen, Riikka-Leena Leskelä, Tea Lallukka, Anna Maria Heikkinen

**Affiliations:** a Department of Public Health, University of Helsinki, Finland; b Kalasatama Health Care Center, City of Helsinki, Finland; c Finnish Institute for Health and Welfare; d Faculty of Medicine and Health Technology, Tampere University; e Wellbeing Services County of Pirkanmaa; f Nordic Healthcare Group, Helsinki, Finland

**Keywords:** Frequent attender, primary health care, general practitioner, nurse, diagnosis, multimorbidity, chronic skin wounds

## Abstract

**Aim:**

This study investigated frequent attenders (FAs) in primary health care in Helsinki, Finland, focusing on their service use, sociodemographic characteristics (sex, age, and language), and diagnostic profiles using registry data.

**Methods:**

Register-based cohort data were drawn from administrative records in primary, specialised, and oral health care of the City of Helsinki and Helsinki University Hospital (2015–2019; *n* = 297 845). FAs were defined as the top decile of annual primary health care users. Physician and nurse face-to-face visits were included. Patients were categorised by how many years (1–5) they met FA criteria. Statistical analyses were performed using the χ^2^ test or, when appropriate, Poisson regression with robust variance estimation to estimate prevalence ratios (PRs).

**Results:**

Frequent attenders had more than seven annual face-to-face primary health care visits. Although representing only 15.9% of patients, FAs accounted for nearly half of all health care visits. FAs were more often women, aged over 65, and native Finnish speakers. Chronic diseases and multimorbidity were more prevalent among FAs. Chronic skin wounds were strongly associated with frequent attendance. As attendance persisted, visit distribution shifted: physician visits increased from 0.7 among non-FAs to 6.4 among 5-year FAs, while nurse visits rose from 0.6 to 11.4 annually, comprising nearly two-thirds of contacts among persistent FAs.

**Conclusion:**

FAs represent a small but high-need group in primary health care. Their service use is driven not only by physicians but disproportionately by nurses, highlighting the importance of including nursing care in research and resource planning.

## Introduction

A large number of health care visits are attributable to a small number of patients, which places a considerable burden on health and social care services. The top decile of frequent attenders (FA) account for 30–50% of all primary health care visits [1–5]. FAs receive five times as many prescriptions and are referred to hospitals with five-fold frequency compared to non-FAs [1]. In a Dutch study [6], the FAs of primary health care incurred three times the costs from specialised care. The difference was not attributed to multimorbidity or to variations in primary care provider practices. A previous study in the Finnish City of Oulu showed that 10% of patients accounted for 81% of all health and social care costs when home care, nursing home and assisted living services were included. The greatest share of cost derived from specialised care or elderly care. The costliest patients utilised services from four different service categories (e.g. primary care, different specialties, different social services), whereas the rest of the population accessed services from only one category on average [7].

Previous studies have shown that the most frequent attenders constitute a diverse group with varying somatic, psychiatric, psychological, and social characteristics. Frequent utilisation has been associated with older age [3,8], the female sex [1,2,4,8–10], chronic disease [2,3,9,11–13], medically unexplained symptoms [3,14], stressful life events [12], anxiety [3,13,15], low socioeconomic position [11], lower educational levels [11] and unemployment [1,2,4,9,12,16]. Only one out of seven patients was found to remain a FA for three consecutive years [3]. These persistent frequent attenders (pFA) exhibit more chronic somatic diseases, psychiatric and social problems, and medically unexplained symptoms than 1-year frequent attendees [3,13]. They also received more prescriptions for psychotropic medication [3].

The need to optimise the management of FAs has been long recognised. Thus far, interventions directed at FAs have proven unconvincing [17,18]. While integrated services and patient-centred care models have been suggested as possible solutions, evidence of their efficacy remains scarce. In OECD countries, the sustainability of primary health care is increasingly threatened by an aging population, rising health care demands, and limited resources. Consequently, understanding the health care and service costs accumulated by FAs is of growing importance.

Most studies on frequent attendance in primary health care have focused exclusively on physician visits. However, in recent years, nurses have assumed a more significant role in primary health care. The substitution of nurses for physicians has been proposed as a strategy to enhance access, efficiency, and quality of care [19]. In Finland, nurses have long played a key role in managing chronic diseases, and many primary health care providers have adopted models whereby nurses serve as the initial point of contact with patients while physicians act primarily as consultants [20]. Given this shift in care delivery, we considered it essential to include all nurse visits to gain a comprehensive understanding of the burden of frequent attendance on primary health care services.

The aim of this study was to examine frequent attenders (FAs) in primary health care in Finland, with a focus on the frequency of service use, sociodemographic characteristics, and diagnostic profiles. First, we studied the frequency of primary health care use among adults living in Helsinki, the Capital of Finland, from 2015 to 2019. Second, we examined certain sociodemographic characteristics of FAs including sex, age and first language. Finally, by integrating registry data from multiple health care services, we developed a more comprehensive description of the diagnoses of primary health care attendees than has been previously possible. Potential multimorbidity of these patients was also assessed.

## Materials and methods

### Study setting

In Finland, primary health care services are structured across three distinct sectors: the public system, private practice, and mostly private occupational health care providers. The Finnish public health and social care system is financed through tax revenue and is universally accessible to all residents of the country.

Preceding the 2022 Social and Health Care reform and throughout our study period, municipalities were responsible for organising and financing public primary health care, oral health care, and social services in Finland. Municipal-run health care centres were responsible for delivering public primary health care services. Conversely, specialised care was predominantly organised and provided by hospital districts formed by regional municipalities, with a smaller proportion of specialised care provided directly by municipalities themselves.

Private health services complement public services. Private primary health care visits are partially covered by the Social Insurance Institution of Finland. In 2019, 31% of Helsinki’s population received partial compensation for private health services [21]. The majority of Finland’s working population does not rely on municipality-run health centres for their primary health care needs [22]. Instead, many employers offer private health care, which includes primary health care services alongside preventative occupational health care as required by Finnish law. When specialist care is needed, most patients are referred to public hospitals by both private and occupational care clinics. In 2023, occupational health care services and private practitioners accounted for roughly 40% of all primary outpatient care in Central-Finland [23].

### Data source

This study is a retrospective cohort study utilizing medical record data extracted from electronic medical records maintained by the City of Helsinki and Helsinki University Hospital between January 2015 and March 2021. The dataset combines electronic records from public primary, specialty, and oral health care. Both patient data (age, sex, diagnosis, first language) and primary health care visit history were examined for this study.

As pandemics strongly affect primary health care systems [24], data from 2020 and 2021 was excluded to minimize the potential effects of the COVID-19 pandemic on Finnish primary health care use. The study population was also restricted to individuals aged 18 years or older at baseline, who resided continuously in Helsinki from 2015 to 2019 and during this time and had at least one face-to-face visit with a primary health care physician or nurse. After applying exclusions, the final study population consisted of 297 845 individuals.

### Definition of frequent attendance in primary health care

Frequent attenders were defined as patients in the top decile of annual primary health care visits, consistent with prior studies [17,25,26]. Frequent attendance was determined separately for each year of the study period (2015–2019) based on the distribution of visits in the study population.

**Figure 1.**
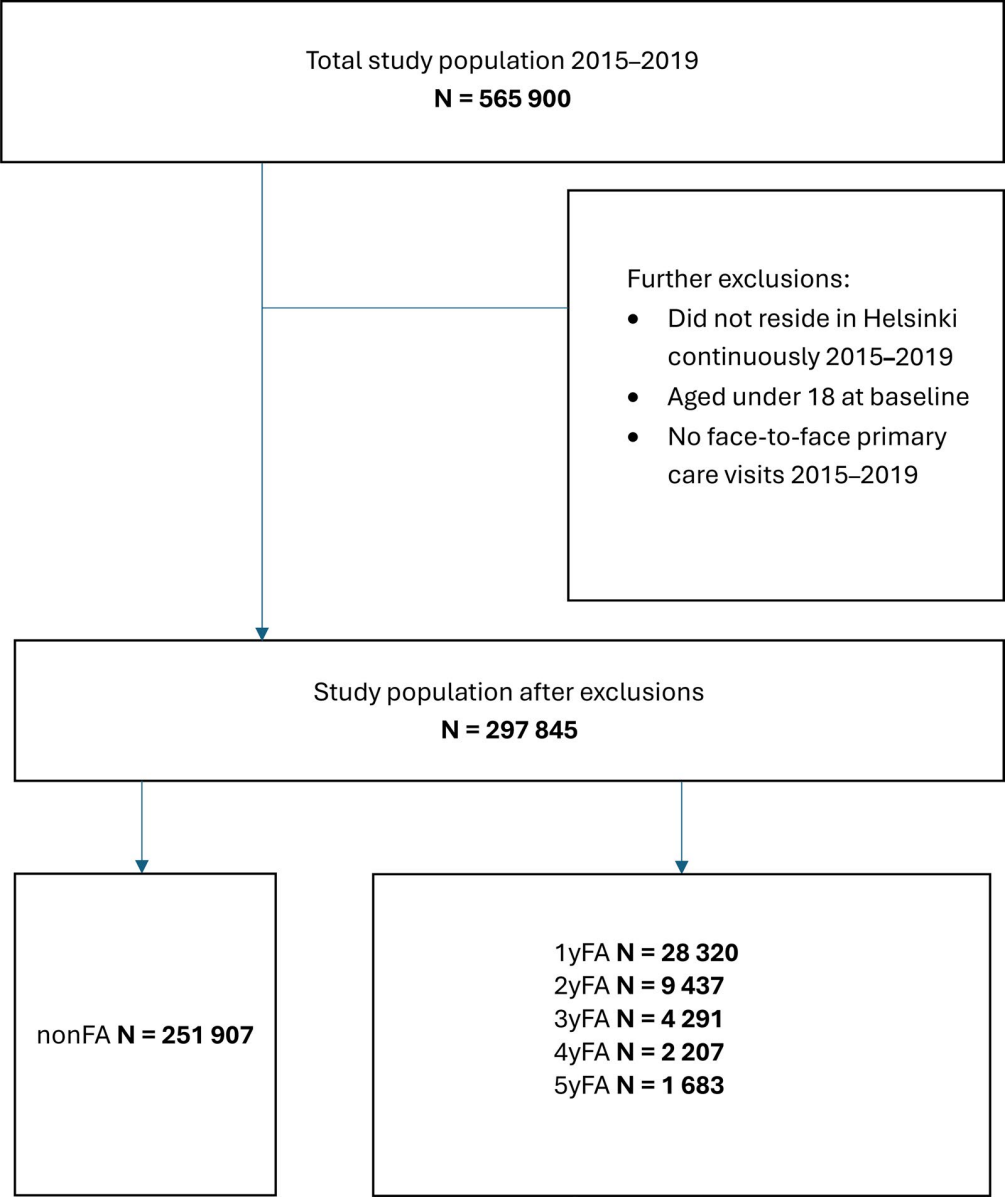
Flowchart depicting the formation of the study population and cohorts. The cohorts were defined as follows: Non-FA: Individuals who were never in the top decile of annual attenders during 2015–2019. 1yFA: Individuals who ranked in the top decile of annual attenders once during 2015–2019. 2yFA: Individuals who ranked in the top decile of annual attenders twice during 2015–2019. 3yFA: Individuals who ranked in the top decile of annual attenders three times during 2015–2019. 4yFA: Individuals who ranked in the top decile of annual attenders four times during 2015–2019. 5yFA: Individuals who ranked in the top decile of annual attenders five times during 2015–2019.

The definition of persistent frequent attendance has varied across studies. Following the approach of Reho et al. [27], we categorised patients based on the frequency with which they met the criteria for frequent attendance during the 5-year study period (Figure 1). Patients who ranked in the top decile of attendance in any one year were classified as 1-year frequent attenders (1yFA). Those who met this criterion in any two years were labelled 2-year frequent attenders (2yFA). Similarly, patients who qualified in three, four, or five years were designated as 3yFA, 4yFA, and 5yFA, respectively. Patients who had at least one primary health care visit between 2015 and 2019 but were never in the top decile of attenders in any year were classified as non-frequent attenders (non-FA).

### Sociodemographic characteristics

The study population was grouped by sex and age (18 to 29, 30 to 49, 50 to 64, 65 years or over) as of 2015. The first language of patients was also examined.

### Diagnoses

Diseases were classified based on ICD-10 (International Statistical Classification of Diseases and Related Health Problems, 10th Revision) using the classification system of the Finnish Institute for Health and Welfare [28].

All recorded ICD-10 diagnosis from primary, specialty, and oral health care were included to represent chronic diseases. ICD-10 diagnostic codes have been registered in the electronic medical records upon physician and nurse visits both in outpatient and inpatient settings using an accuracy of three digits. ICPC-2 codes were not available in our dataset. In Finnish primary health care, physicians primarily code visits using ICD-10. Nurses typically use ICPC-2 codes, but in cases where a patient already has an existing ICD-10 diagnosis recorded by a physician, nurses are encouraged to use the ICD-10 code instead of ICPC-2 [29]. The Finnish Institute for Health and Welfare manage and track diagnosis data registration. According to their records, the ICD-10 coding rates for all primary health care physician visits in Helsinki in 2019 was 86% [30]. Among nurse visits, the overall diagnosis recording rate was 85%, with 20% of visits coded using ICD-10 and 67% coded using ICPC-2 [30].

The Charlson Comorbidity Index (CCI) was used to assess comorbidity [31,32]. The CCI includes a list of 19 medical conditions, each assigned a weighted score based on its impact on mortality. A score of 0 points in the CCI indicates no comorbid conditions and or a low risk of mortality; 1–2 points indicate mild comorbidity with a moderate risk; 3–4 points indicate moderate comorbidity with a substantial risk; and 5 or more points indicate severe comorbidity with a high risk of mortality.

### Statistical analyses

Statistical analyses were performed using the χ^2^ test or, when appropriate, Poisson regression with robust variance estimation to estimate prevalence ratios (PRs) and their 95% confidence intervals, following the methods described by Yorlets [33] and Barros & Hirakata [34]. The PR was utilised due to the substantial number of cases across different diagnostic groups [35]. The prevalence ratio was adjusted for age and sex.

All analyses were conducted using R Statistical Software (version 4.3.1; R Core Team, 2023). A p-value < 0.05 was considered statistically significant.

### Ethical considerations

Research permission for our study plan was granted by the Finnish Social and Health Data Permit Authority Findata (THL/6607/14.05.00/2021and THL/5969/14.06.00/2022). Data from different registers were collected by Findata in pseudonymised form. All statistical analyses were conducted in Findata′s secure operating environment Kapseli^®^. The ethical board of the Medical Faculty of the University of Helsinki gave an affirmative statement of this study plan on the 7^th^ of December 2021 (No. 19/2021).

## Results

When applying the top 10^th^ percentile threshold for frequent attendance, individuals with >7 annual face-to-face primary health care visits were classified as frequent attenders (FAs) for that year. Individuals with ≤7 annual visits were classified as non-FAs. Overall, 15.4% of the study population were classified as FAs at least once during 2015–2019, yet they accounted for nearly half of all visits (Table 1). A small proportion (0.6%) were frequent attenders every year (5yFAs), but this group alone contributed almost 150,000 visits, disproportionately adding to the overall burden of primary care.

**Table 1.** Number of in-person visits to primary health care physicians and nurses in Helsinki 2015–2019, by frequent attender (FA) group. A total of 3 106 865 visits among 297 845 individuals.

		Non-FA	1-year-FA	2-year-FA	3-year-FA	4-year-FA	5-year-FA
Individuals							
	n (total 297 845)	251 907	28 320	9 437	4 291	2 207	1 683
Total number of visits							
	n (total 3 106 865)	1 664 219	628 246	327 789	201 883	135 108	149 620
Visits per year							
	Mean	1.3	4.4	6.9	9.4	12.2	17.8
	Standard deviation	1.1	1.6	2.5	4.1	6.6	9.4
	Median	1.0	4.4	6.6	8.6	10.6	15.0
Physician visits							
	n	919 496	321 547	162 814	96 636	60 925	53 629
	% all visits	55.3%	51.2%	49.7%	47.9%	45.1%	35.8%
Physician visits per year							
	Mean	0.7	2.3	3.5	4.5	5.5	6.4
	Standard deviation	0.7	1.1	1.3	1.7	2.3	4.1
	Median	0.6	2.2	3.4	4.6	5.4	6.4
Nurse visits							
	n	744 723	306 699	164 975	105 247	74 183	95 991
	% all visits	44.7%	48.8%	50.3%	52.1%	54.9%	64.2%
Nurse visits per year							
	Mean	0.6	2.2	3.5	4.9	6.7	11.4
	Standard deviation	0.6	1.4	2.6	4.4	6.9	10.1
	Median	0.4	2.0	3.0	3.8	5.0	8.0

1-year-FA: Individuals ranking in the top decile of annual attenders once during 2015–2019.

2-year-FA: Individuals ranking in the top decile of annual attenders twice during 2015–2019.

3-year-FA: Individuals ranking in the top decile of annual attenders three times during 2015–2019.

4-year-FA: Individuals ranking in the top decile of annual attenders four times during 2015–2019.

5-year-FA: Individuals ranking in the top decile of annual attenders five times during 2015–2019.

Non-FA: Reference group consisted of individuals that were never among the top decile of annual attenders.

The intensity of health care use increased progressively with the persistence of frequent attendance. On average, non-FAs had 1.3 visits per year, while 5yFAs had 17.8 visits annually – more than 15 times higher. Median visit counts followed the same trend, rising from 4.4 visits/year in the 1yFA group to 15 visits/year in the 5yFA group, compared with a median of 1 visit/year in non-FAs.

When examining visit types, physician visits were more common among non-FAs, accounting for 55.3% of all encounters, while nurse visits made up 44.7%. However, this distribution shifted steadily as the persistence of frequent attendance increased. Among 5yFAs, nearly two-thirds of visits (64.2%) were nurse contacts, while physician encounters decreased proportionally to 35.8%. The mean annual number of physician visits rose from 0.7 among non-FAs to 6.4 among 5yFAs, while nurse visits increased even more sharply, from 0.6 to 11.4 per year. Thus, the higher total visit burden among persistent FAs was driven primarily by an accumulation of nurse visits, although physician visits also increased substantially.

Frequent attenders were more often women, representing 62.0% of 1yFAs and increasing to 70.3% in the 4yFA group, before slightly declining to 66.3% in 5yFAs (Table 2). The proportion of older adults (≥65 years) also increased with FA duration, from 40.9% in 1yFAs to 55.3% in 5yFAs, compared with 21.8% in non-FAs. Native Finnish speakers were overrepresented among frequent attenders, particularly in the 5yFA group (88.0%) compared to other languages. All differences in sex, age, and language distribution between FA groups and non-FAs were statistically significant (χ^2^ test, *p* < 0.001).

**Table 2.** Sex, age and first language of frequent attenders (FA) of primary health care in Helsinki 2015–2019 (*N* = 297 845).

		Non-FA		1-year-FA		2-year-FA		3-year-FA		4-year-FA		5-year-FA	
		n	%	n	%	n	%	n	%	n	%	n	%
Sex													
	Male	106 724	42.4	10 772	38.0	3 295	34.9	1 409	32.8	655	29.7	568	33.7
	Female	145 183	57.6	17 548	62.0	6 142	65.1	2 882	67.2	1 552	70.3	1 115	66.3
Age													
	18–29	53 835	21.4	4 067	14.4	1 006	10.7	336	7.8	136	6.2	51	3.0
	30–49	85 046	33.8	6 480	22.9	1 923	20.4	830	19.3	406	18.4	280	16.6
	50–64	58 102	23.1	6 182	21.8	1 958	20.7	853	19.9	436	19.8	421	25.0
	65–	54 924	21.8	11 591	40.9	4 550	48.2	2 272	52.9	1 229	55.7	931	55.3
Language													
	Finnish	209 391	83.1	23 384	82.6	7 971	84.5	3 620	84.4	1 886	85.5	1 481	88.0
	Swedish	13 364	5.3	1 295	4.6	386	4.1	189	4.4	80	3.6	68	4.0
	Russian	7 123	2.8	830	2.9	243	2.6	84	2.0	56	2.5	36	2.1
	Estonian	3 515	1.4	318	1.1	80	0.8	23	0.5	11	0.5	3	0.2
	Somali	2 515	1.0	492	1.7	166	1.8	87	2.0	48	2.2	20	1.2
	English	1 775	0.7	166	0.6	44	0.5	20	0.5	6	0.3	6	0.4
	Arabic	1 520	0.6	274	1.0	100	1.1	47	1.1	22	1.0	13	0.8
	Other	12 704	5.0	1 561	5.5	447	4.7	221	5.2	98	4.4	56	3.3

The p-value, based on the χ^2^ test, was **< 0.001** for sex, age and language in each FA group (reference: non-FA).

From 2015 to 2019, the diagnosis recording rate using ICD-10 was 88.8% for physician visits and 20.7% for nurse visits (Table in appendix). Chronic disease prevalence was strongly associated with frequent attendance (Table 3). Chronic skin wounds showed the strongest association, with age- and sex-adjusted prevalence ratios (PR) ranging from 4.28 in 1yFAs to 9.44 in 5yFAs. Other conditions with pronounced associations included psychotic and bipolar disorders (PR 1.90–8.49), personality disorders (PR 2.81–7.41), migraine and other headaches (PR 2.55–6.51), and obesity (PR 2.33–5.01). Common chronic conditions such as hypertension, diabetes, and depression/anxiety disorders were also strongly associated with frequent attendance. Age- and sex-adjusted prevalence ratios increased progressively with the frequency of attendance: for hypertension, PR ranged from 1.36 in 1yFAs to 1.56 in 5yFAs; for diabetes, PR increased from 1.64 to 2.51; and for depression and anxiety disorders, PR rose from 2.14 to 3.67. Oral health diseases were also more common, with dental caries and periodontal diseases showing higher prevalence ratios among frequent attenders, increasing steadily with the number of years classified as a FA (Tables 4 and 5).

**Table 3.** Association between chronic diseases and frequent attendance (FA) 2015–2019, based on age and sex adjusted prevalence ratio (PR).

	Non-FA (ref.)	1-year-FA		2-year-FA		3-year-FA		4-year-FA		5-year-FA	
	n	n	PR (95% CI)	n	PR (95% CI)	n	PR (95% CI)	n	PR (95% CI)	n	PR (95% CI)
Alcohol and drug-related disorders	7 909	1 838	2.26 (2.15–2.38)	699	2.72 (2.52–2.93)	359	3.18 (2.87–3.53)	181	3.26 (2.83–3.76)	127	2.87 (2.42–3.40)
Asthma	11 407	3 471	2.42 (2.33–2.51)	1 712	3.41 (3.25–3.57)	901	3.81 (3.58–4.06)	577	4.62 (4.29–4.97)	463	4.88 (4.50–5.28)
Atrial fibrillation	10 901	3 198	1.51 (1.46–1.57)	1 506	1.79 (1.71–1.88)	814	1.92 (1.81–2.05)	455	2.02 (1.86–2.18)	355	2.07 (1.89–2.26)
Back pain and spinal cord disorders	39 871	10 867	2.26 (2.22–2.30)	4 947	2.99 (2.93–3.06)	2 585	3.37 (3.28–3.46)	1 487	3.71 (3.59–3.83)	1 049	3.42 (3.29–3.56)
COPD	3 830	1 412	2.34 (2.20–2.49)	728	3.24 (3.00–3.51)	396	3.62 (3.27–4.01)	225	3.91 (3.43–4.47)	220	4.92 (4.31–5.61)
Cancer	16 675	4 261	1.49 (1.45–1.54)	1 717	1.56 (1.49–1.63)	937	1.71 (1.62–1.81)	506	1.72 (1.59–1.85)	377	1.68 (1.54–1.83)
Cerebrovascular diseases	9 673	2 449	1.37 (1.31–1.42)	1 072	1.52 (1.44–1.62)	552	1.56 (1.44–1.69)	323	1.70 (1.54–1.87)	265	1.83 (1.64–2.04)
Chronic skin wounds	1 570	1 110	4.28 (3.95–4.63)	600	6.12 (5.56–6.74)	382	7.93 (7.09–8.87)	238	9.37 (8.19–10.73)	186	9.44 (8.14–10.96)
Coronary artery disease	9 559	2 638	1.48 (1.42–1.54)	1 237	1.78 (1.68–1.88)	642	1.85 (1.72–1.99)	384	2.11 (1.93–2.30)	288	2.05 (1.84–2.28)
Dementia	6 512	1 662	1.14 (1.08–1.20)	676	1.12 (1.04–1.20)	373	1.19 (1.08–1.31)	208	1.19 (1.05–1.35)	121	0.95 (0.80–1.13)
Dental caries	90 747	13 710	1.42 (1.40–1.44)	5 118	1.63 (1.60–1.66)	2 436	1.74 (1.69–1.78)	1 315	1.84 (1.78–1.91)	1 002	1.86 (1.78–1.93)
Depression and anxiety disorders	31 188	6 394	2.14 (2.09–2.19)	2 533	2.75 (2.66–2.84)	1 294	3.27 (3.13–3.42)	662	3.36 (3.16–3.58)	515	3.67 (3.42–3.94)
Diabetes mellitus	20 881	5 213	1.64 (1.59–1.69)	2 251	1.92 (1.85–2.00)	1 168	2.06 (1.95–2.17)	691	2.32 (2.17–2.48)	582	2.51 (2.33–2.69)
Disorders of bone density and structure	2 890	903	1.63 (1.52–1.76)	440	1.97 (1.79–2.17)	248	2.15 (1.90–2.44)	153	2.33 (1.99–2.71)	146	3.03 (2.60–3.54)
Dissociative and somatization disorders	5 936	1 143	1.98 (1.86–2.11)	462	2.57 (2.35–2.82)	242	3.13 (2.76–3.54)	147	3.78 (3.23–4.42)	139	5.16 (4.41–6.05)
Heart failure	4 038	1 494	1.82 (1.72–1.93)	773	2.34 (2.17–2.51)	462	2.74 (2.51–3.00)	268	2.93 (2.62–3.28)	221	3.23 (2.85–3.65)
Hypertension	47 534	10 884	1.36 (1.34–1.38)	4 483	1.46 (1.43–1.50)	2 338	1.54 (1.50–1.58)	1 291	1.58 (1.52–1.64)	976	1.56 (1.50–1.63)
Kidney failure and chronic renal diseases	6 088	1 697	1.76 (1.67–1.86)	782	2.16 (2.01–2.33)	474	2.67 (2.45–2.92)	243	2.57 (2.27–2.90)	209	2.87 (2.52–3.26)
Migraine and other headache syndromes	9 466	2 463	2.55 (2.45–2.66)	1 163	3.77 (3.56–3.99)	648	4.77 (4.44–5.13)	390	5.63 (5.15–6.16)	319	6.51 (5.90–7.18)
Obesity	6 408	1 778	2.33 (2.21–2.46)	862	3.31 (3.09–3.55)	457	3.80 (3.46–4.17)	278	4.45 (3.96–4.99)	240	5.01 (4.43–5.66)
Osteoarthritis	3 012	634	1.53 (1.41–1.67)	326	2.18 (1.94–2.44)	160	2.22 (1.90–2.60)	108	2.80 (2.31–3.38)	110	3.74 (3.11–4.51)
Other atherosclerotic diseases	2 414	918	2.07 (1.92–2.23)	464	2.69 (2.44–2.97)	275	3.19 (2.83–3.61)	152	3.34 (2.85–3.92)	136	3.89 (3.30–4.59)
Other sleep disorders	11 017	3 014	2.34 (2.25–2.43)	1 376	3.14 (2.98–3.32)	726	3.60 (3.36–3.86)	421	4.02 (3.67–4.39)	341	4.28 (3.88–4.72)
Periodontal disease	44 952	7 324	1.52 (1.49–1.56)	2 812	1.79 (1.74–1.85)	1 385	1.97 (1.89–2.06)	800	2.23 (2.11–2.36)	570	2.11 (1.97–2.26)
Personality disorders	2 487	606	2.81 (2.58–3.07)	268	4.31 (3.81–4.88)	137	5.45 (4.60–6.45)	82	6.84 (5.52–8.46)	58	7.41 (5.75–9.56)
Psychotic and bipolar disorders	6 989	1 341	1.90 (1.79–2.01)	598	2.67 (2.46–2.90)	336	3.43 (3.08–3.81)	242	4.92 (4.36–5.56)	315	8.49 (7.67–9.41)
Rheumatoid arthritis and other inflammatory polyarthropaties	20 369	5 725	1.70 (1.66–1.75)	2 819	2.19 (2.11–2.26)	1 519	2.37 (2.27–2.47)	877	2.51 (2.38–2.65)	692	2.61 (2.46–2.77)
Sleep apnea	7 346	1 614	1.78 (1.69–1.88)	762	2.47 (2.30–2.66)	389	2.73 (2.48–3.02)	235	3.25 (2.87–3.68)	183	3.18 (2.77–3.67)

The age- and sex-adjusted prevalence ratio (PR) was calculated using a Poisson regression model with robust variance estimation. The p-value, based on the Wald test, was < 0.001 for the prevalence ratio in each FA group (reference: non-FA) across all diseases, except for dementia, where the p-value was 0.58 in the 5-year-FA group, 0.006 in the 4-year-FA group and 0.004 in the 2-year-FA group. Diagnoses were categorized according to the disease classification system of the Finnish Institute for Health and Welfare based on ICD-10 codes. COPD = chronic obstructive pulmonary disease.

**Table 4.** The association with the most common disease groups and frequent attendance (FA), based on crude prevalence.

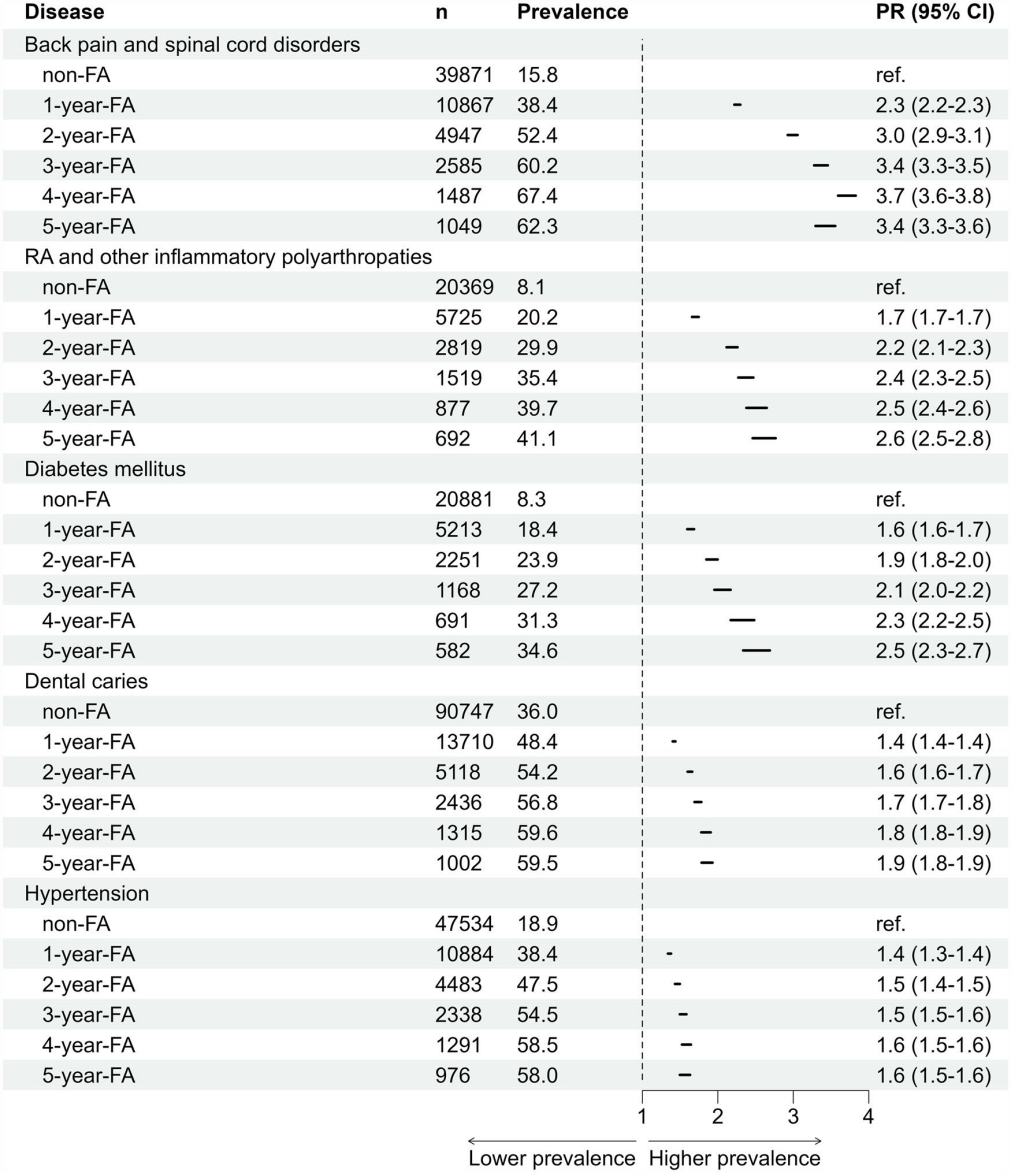

PR = age and sex adjusted prevalence ratio.

RA = Rheumatoid arthritis.

**Table 5.** The disease groups with the strongest association with frequent attendance (FA), based on age and sexadjusted prevalence ratio (PR).

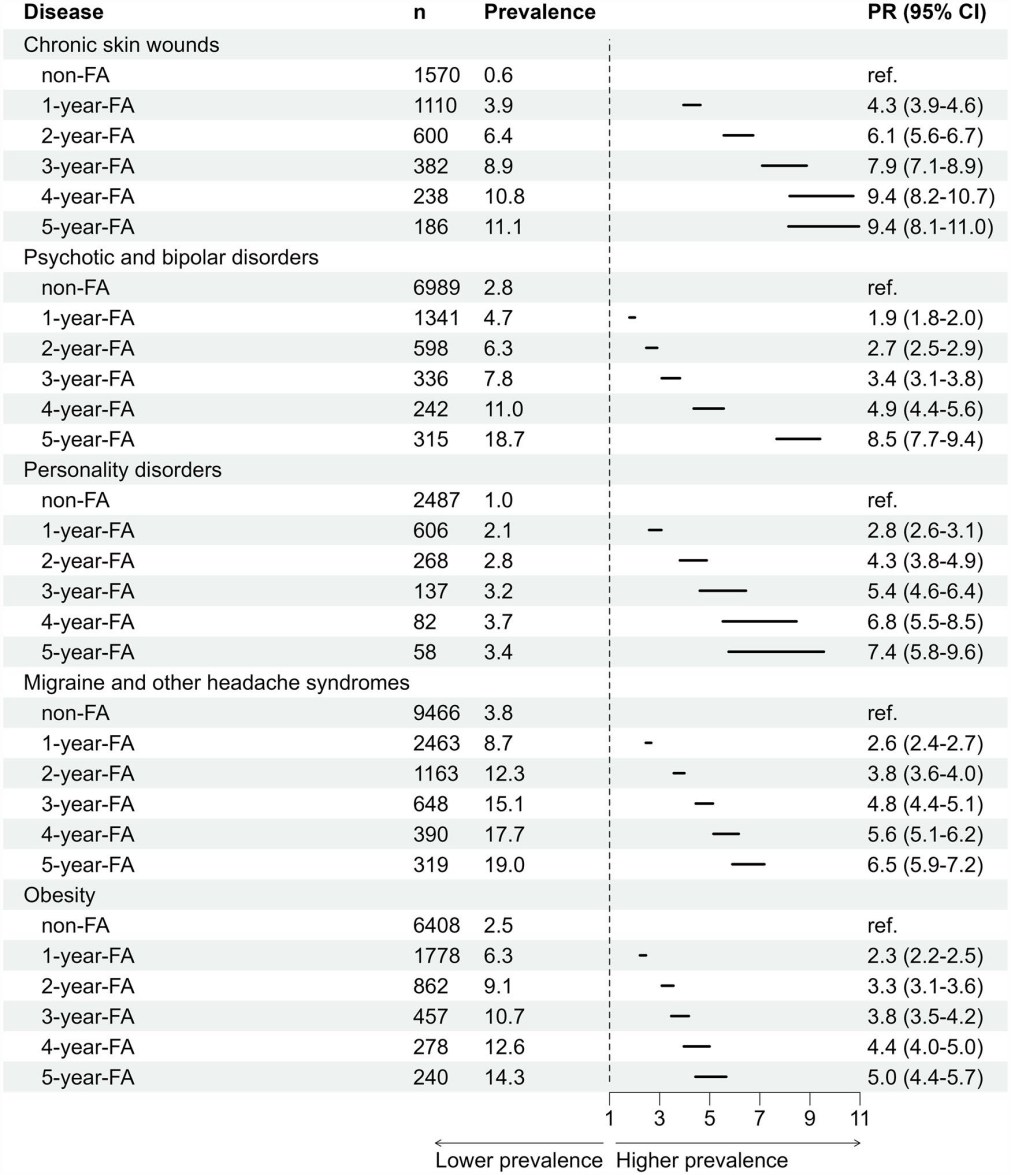

Multimorbidity, measured using the Charlson Comorbidity Index (CCI), increased with the frequency of attendance (Table 6). In the 5yFA group, 28.3% had no comorbidities, compared with 74.7% in non-FAs. A total of 16.5% of 5yFAs had moderate comorbidity (CCI 3–4) and 3.5% had severe comorbidity (CCI ≥5), while only 1.7% of non-FAs had moderate comorbidity, and 0.1% had severe comorbidity. These results indicate that frequent attendance is strongly linked to the having multiple chronic conditions.

**Table 6.** Comorbidity among frequent attender (FA) cohorts using Charlson Comorbidity Index (CCI) 2015–2019.

		Non-FA		1-year-FA		2-year-FA		3-year-FA		4-year-FA		5-year-FA	
		n	%	n	%	n	%	n	%	n	%	n	%
Charlson Comorbidity Index (CCI)													
	0	188 178	74.7	15 335	54.1	3 971	42.1	1 534	35.7	618	28.0	476	28.3
	1–2	50 213	19.9	11 054	39.0	4 454	47.2	2 147	50.0	1 185	53.7	870	51.7
	3–4	4 161	1.7	1 733	6.1	875	9.3	520	12.1	335	15.2	277	16.5
	5+	345	0.1	187	0.7	136	1.4	90	2.1	69	3.1	59	3.5

The p-value, based on the χ^2^ test, was **< 0.001** for the Charlson Comorbidity Index in each FA group (reference: non-FA). The severity of comorbidity, as measured with CCI, was categorized into four levels.

A score of 0 points in the CCI indicates no comorbid conditions and or a low risk of mortality; 1–2 points indicate mild comorbidity with a moderate risk; 3–4 points indicate moderate comorbidity with a substantial risk; and 5 or more points indicate severe comorbidity with a high risk of mortality.

## Discussion

### Main findings

We found that a small portion of patients (15.9%) accounted for nearly half of all primary care visits during our 5-year study period. Frequent attenders were more often women, over 65 years of age, and native Finnish speakers. Furthermore, chronic diseases were more prevalent among frequent attenders, with chronic skin wounds standing out as a diagnosis significantly associated with frequent attendance. Multimorbidity among frequent attenders was common. Frequent service use includes visits to both ­physicians and nurses, with nursing encounters accounting for a considerable proportion.

### Interpretation

#### Demographic patterns

Our findings are consistent with previous studies showing that frequent attendance in primary health care is more common among women [1,2,4,8–10,36,37] and older adults [3,8]. While higher multimorbidity prevalence among women and the elderly in Finland [38] partly explains this pattern, earlier research also highlights the role of health-seeking behaviour as a contributing factor [39–41]. Women, for instance, are generally more proactive in consulting health services, more likely to seek care at earlier stages of illness, and more inclined to use preventive and follow-up services compared to men [39,40]. Similarly, older adults often demonstrate lower thresholds for consulting primary care due to increased health concerns [41].

Native Finnish-speaking patients were more likely to become frequent attenders of primary health care than Swedish- or foreign-language speakers. Previous studies have shown that Swedish-speaking Finns have a higher life expectancy than Finnish-speaking Finns, particularly among men [42]. This suggests that Swedish-speaking individuals may be healthier. The lack of language-concordant communication may also reduce patients’ willingness to seek care and contribute to lower utilisation [43,44].

Migrant-background patients, defined here as having a mother tongue other than Finnish or Swedish, also appeared underrepresented among frequent attenders. This aligns with international evidence [45,46] showing that immigrants often use fewer health services despite high health needs, largely due to barriers in communication, continuity of care, and system navigation. These findings should therefore be interpreted cautiously. Furthermore, people with a migrant background tend to be younger on average [47]. This may also contribute to their lower apparent use of health care services.

#### Clinical patterns

Including nurse visits revealed a broader understanding of frequent attenders’ overall use of primary health care in Finland. Our findings demonstrate a clear shift in care provision as frequent attendance persists. While physician visits increased with attendance frequency, nurse visits rose even more sharply. Among patients who were persistent frequent attenders over five years, nearly two-thirds of visits were with nurses, suggesting that nurses play a central role in managing the ongoing healthcare needs of this group. This pattern likely reflects nurses’ involvement in chronic disease management, follow-up care, patient education, and care coordination, underscoring the importance of nurse-led interventions in addressing the healthcare demands of frequent attenders. The growing reliance on nurses for both routine and complex care highlights the need to ensure sufficient nursing resources and support in primary care.

Multimorbidity, as indicated by the Charlson Comorbidity Index (CCI), was associated with becoming a persistent frequent attender. This aligns with previous findings suggesting that the use of primary health care services increases with multimorbidity, regardless of the index or measure used [48]. Therefore, multimorbid patients should always be considered potential future pFA patients. In Finland, multimorbidity affects roughly one-third of adults, with musculoskeletal and cardiometabolic conditions representing the most common and costly clusters [38]. Given limited resources, providing continuity of care for all multimorbid patients may not be feasible. Instead, prioritising subgroups with high complexity, heavy service use, and high risk of adverse outcomes – such as patients with combined cardiometabolic and mental health conditions – may be a pragmatic approach.

The strong association between chronic skin wounds and frequent attendance in primary health care has not been prominently highlighted in previous studies, likely due to their emphasis on physician visits. Although the high health care needs of patients with chronic wounds are clinically well recognised, their contribution to frequent attendance and nursing workload may be underappreciated in the literature. In developed countries, chronic wounds (e.g. pressure ulcers, diabetic foot ulcers, and venous leg ulcers) are estimated to account for 2–4% of total health care costs [49] with nursing time and hospital costs together responsible for around 80–85% of the total cost [50]. Nussbaum et al. [51] suggested that the morbidity and associated costs of chronic wounds are largely overlooked in public policy, possibly because no specific medical specialty is explicitly designated as responsible for wound care. Our findings demonstrate that patients with chronic skin wounds represent a distinct subgroup of frequent attenders characterised by a high volume of nurse visits, underscoring the central role of nursing care in wound management within public primary health care in Finland. The observed association is clinically, and economically significant, as chronic skin wounds typically require frequent monitoring, dressing changes, and long-term follow-up, all of which contribute to sustained primary care utilisation. In Helsinki, a specialised wound care team was established in 2013; however, previous research has shown no reduction in the prevalence of chronic wounds between 2008 and 2016, possibly reflecting challenges in guideline implementation [52]. While centralised wound care units may support standardisation and resource optimisation, our findings highlight the continued importance of prevention, early identification, and effective long-term management of chronic wounds within primary care, particularly among high-risk and multimorbid populations.

Psychotic and bipolar disorders, personality disorders, and depression and anxiety disorders, were strongly associated with frequent attendance in our study. This is consistent with prior research demonstrating that psychiatric morbidity, psychological distress, and other psychosocial factors are strongly associated with frequent attendance in primary care, often reflecting complex and long-term care needs related to the coexistence of psychiatric and somatic comorbidities and unmet psychosocial support needs [3,13,15,41,53–55]. While a recent meta-analysis identified depression, anxiety, and somatization as the most common psychological diagnoses among frequent attenders [53], our findings suggest that a broader spectrum of severe mental disorders is also relevant.

Frequent primary care utilisation may occur particularly when access to appropriate basic-level psychiatric support services – such as brief psychotherapeutic interventions or timely crisis support – and specialised mental health services is limited. Insufficient access to appropriate services may contribute to the persistence or worsening of psychiatric conditions, despite evidence that early intervention improves recovery and long-term outcomes [56,57]. These findings highlight the importance of integrated and continuous care models within primary health care, especially for patients with severe or persistent mental health conditions, which may reduce avoidable frequent attendance.

Systematic reviews [9,25] of the frequent attendance literature typically focus on need factors such as chronic physical illness and mental health, but do not include oral health service use, highlighting a gap in existing research. In the present study, oral diseases including dental caries and periodontal disease were more prevalent among frequent attenders than among non-frequent attenders, with prevalence ratios increasing consistently with the number of years a patient met the frequent attender criteria. Oral health diagnoses thus accumulated in the same patient groups as other chronic conditions, suggesting that oral diseases form part of the overall morbidity burden characteristic of frequent attenders.

These findings appear to contrast with those of a recent Finnish study [58] that reported that among patients with a high volume of service use (≥10 visits), concurrent intensive utilization of both public oral health services and public health care services was rare, occurring in only five percent of cases. However, access to oral health care in Finland is socially patterned, with higher-income individuals more likely to use services, primarily in the private sector [59]. As data on private oral health care utilization were unavailable, the observed associations between frequent attendance and oral health service use may be underestimated and could differ if private services were included [60].

Evidence from studies outside the frequent-attender literature indicates that regular use of oral health care services is associated with better oral health outcomes, underscoring the importance of considering oral health as part of comprehensive patient assessment in primary care [61]. For example, a recent register-based study conducted in the city of Helsinki [62] reported poorer periodontal outcomes among patients with diabetes and severe mental disorders – groups that, according to both the present study and previous research represent typical frequent attenders. These findings may suggest insufficient utilization of oral health care services among patients with diabetes and/or severe mental disorders, particularly among those with oral diseases requiring special attention and integrated care. Further research is warranted to better understand the role of oral health in frequent attendance and its implications for integrated primary care.

### Strengths and limitations

Notable strengths of our study are its large and comprehensive dataset, including a more comprehensive set of diagnoses, and a long follow-up. Moreover, the data used in this study is register-based and therefore less prone to bias as compared to, for example, self-reported diagnoses.

A novel feature of our study was the inclusion of primary care nurse visits when evaluating frequent attendance. Including nurse visits not only provided a more complete picture of total service utilisation but also highlighted the disproportionate contribution of nurses to the care of persistent frequent attenders, an aspect often overlooked in previous studies focusing solely on physician visits. While the demographic profile of frequent attenders remained similar, the diagnostic profile became more nuanced, highlighting nursing-intensive conditions that might otherwise be overlooked. This is consistent with international evidence showing that nurses play a central role in the management of chronic diseases, preventive care, and patient education, particularly in primary care settings [19]. These findings underscore the importance of including the full spectrum of primary care providers when assessing utilisation and planning resources.

A limitation of our study is the incomplete diagnostic coding of nurse visits, as ICPC-2 codes were not included in our dataset. While physicians recorded diagnoses for 88.8% of visits using ICD-10 codes, the corresponding rate among nurses was substantially lower, at 20.7%. Consequently, visit-specific diagnostic data from nurse encounters could not be reliably analysed. Instead, we opted to assess patients’ diagnostic profiles over the entire five-year study period, which allowed us to capture chronic and long-term conditions more comprehensively. Although this approach may underestimate the contribution of certain acute or nursing-intensive conditions at the visit level, it provides a more valid basis for identifying multimorbidity and long-term illness patterns among frequent attenders. Accounting for nurse visits still yielded meaningful findings that would have been overlooked if only physician visits had been considered, such as the previously mentioned chronic skin wounds.

Our findings should be interpreted as reflecting associations between long-term morbidity burden and frequent attendance, rather than the immediate clinical reasons for individual physician or nurse visits. Acute, symptom-based encounters such as infections, minor traumas, or short-term complaints could not be reliably assessed, which may influence interpretations of the distribution of visit types between professional groups. Future studies combining visit-level diagnostic data with longitudinal ­morbidity information would be valuable in further clarifying the drivers of frequent attendance in ­primary care.

Additional limitations must be acknowledged. Our data primarily reflects individuals who access municipal health services, receive diagnostic coding, and continue their care within the public system. Consequently, working-age individuals with occupational health coverage and higher-income groups using private services are underrepresented. A significant proportion of the employed working-age population in Finland obtain their primary health care services from occupational health care providers. The providers are predominately private operators and therefore their data could not be included in our study. The working-age patients who do utilise public primary health care services in Finland are frequently unemployed or retired due to disability, which potentially introduces demographic bias. It is also important to acknowledge that registry data, in general, may obscure health inequities, as populations with the greatest unmet healthcare needs often appear underrepresented. Previous studies have shown that socially vulnerable groups – including migrants [63], the homeless [64], individuals facing economic hardship [65], as well as those with severe mental illness [64,66,67], substance use disorders [67], or cognitive impairments [68] – encounter significant barriers to accessing care, discontinuities in follow-up, and challenges in navigating the healthcare system. This underrepresentation may also partly account for the relatively low proportion of foreign-language-speaking patients in our cohort, despite international evidence indicating that migrants frequently have elevated healthcare needs [64,67–69]. Additionally, systemic biases may be present due to the characteristics of our primary health care system. While the Finnish primary health care is, in principle, universally accessible to all residents, several factors hinder access. High co-payments, including those for primary health care services, minimal exemptions, and high annual ceilings on out-of-pocket medicine expenses create financial barriers to care [70]. Moreover, fragmentation in health care financing and service delivery contributes to inequitable access to primary health care in Finland, making the country an outlier among Nordic nations in terms of out-of-pocket and catastrophic health expenditures [70]. Fortunately, Helsinki does not charge co-payments for primary health care visits, making it the only Well-being area in Finland to do so. This can also be considered a strength of our study, as financial barriers do not hinder access to care.

Our data lacked important sociodemographic factors such as education, occupational, and marital status, as well as health behavioural markers such as body mass index and smoking status. Another limitation was the inability to assess the health care costs associated with FAs, which would have provided valuable insights into the resource burden posed by this patient group. Unfortunately, detailed cost data linked to individual health care utilisation in primary care were not available. Further research is needed in this regard.

### Health policy implications and prospects

Frequent users of primary health care should be better identified and provided with personalised, targeted care pathways. Comprehensive health assessments to detect chronic diseases and manage multimorbidity are essential in addressing frequent attendance in primary care. Continuity of care with a trusted physician has consistently been shown to reduce unnecessary visits [71,72], hospitalisations [71] and medicalisation [73,74], as well as lower health care costs [75] and even mortality [76].

However, continuity of care has been declining in Finnish primary care [77,78], partly due to the multi-provider system and the shortage of long-term primary care physicians. Approximately one-third of physicians working in public primary care in Finland are specialists in general practice or long-term primary care providers. The remaining two-thirds are typically physicians in training, often employed on a temporary basis [79]. As a result, universal continuity of care is currently not feasible in Finland. Patient segmentation models have therefore been proposed as a pragmatic strategy for allocating resources to those who stand to benefit most [80,81]. Several providers, including the City of Helsinki, have implemented care pathway models to identify vulnerable patients, who may then receive case management, integrated services, and optimised treatment plans. Multimorbid patients – particularly those with cardiometabolic, musculoskeletal, or chronic skin conditions – should be considered at elevated risk of becoming frequent attenders. Beyond medical management, addressing frequent attendance requires a broader approach. Case management programs, involving multidisciplinary teams could help alleviate this burden [82]. Additionally, as frequent attendance is often linked to mental health issues, stronger integration of mental health services into primary services could be beneficial given the strong association between frequent attendance and psychological distress. Social interventions, such as connecting patients with community resources, may also reduce dependence on primary care for emotional support [83]. Improving the cultural competence of health care providers and addressing language barriers, such as providing access to professional interpreters, can help reduce obstacles to seeking health care [84,85].

The policy debate in Finland on reintroducing a personal doctor model illustrates growing recognition of the importance of continuity. A 2022 initiative [79] commissioned by the Finnish Ministry of Social Affairs and Health, recommended embedding such a model into legislation. While its implementation would require substantial investment in primary care and a significant increase in physician capacity, it represents a potential step toward strengthening continuity of care and addressing the disproportionate burden posed by frequent attenders.

## Data Availability

The data are accessible only within Findata′s secure operating environment Kapseli^®^ to researchers with an approved research permit. Further information can be provided upon reasonable request.

## Declarations

The authors used ChatGpt (Open AI) to assist with language editing. The authors are responsible for all content.
